# Physician supply forecast: better than peering in a crystal ball?

**DOI:** 10.1186/1478-4491-7-10

**Published:** 2009-02-13

**Authors:** Dominique Roberfroid, Christian Leonard, Sabine Stordeur

**Affiliations:** 1 Belgian Health Care Knowledge Centre, Brussels, Belgium

## Abstract

**Background:**

Anticipating physician supply to tackle future health challenges is a crucial but complex task for policy planners. A number of forecasting tools are available, but the methods, advantages and shortcomings of such tools are not straightforward and not always well appraised. Therefore this paper had two objectives: to present a typology of existing forecasting approaches and to analyse the methodology-related issues.

**Methods:**

A literature review was carried out in electronic databases Medline-Ovid, Embase and ERIC. Concrete examples of planning experiences in various countries were analysed.

**Results:**

Four main forecasting approaches were identified. The supply projection approach defines the necessary inflow to maintain or to reach in the future an arbitrary predefined level of service offer. The demand-based approach estimates the quantity of health care services used by the population in the future to project physician requirements. The needs-based approach involves defining and predicting health care deficits so that they can be addressed by an adequate workforce. Benchmarking health systems with similar populations and health profiles is the last approach. These different methods can be combined to perform a gap analysis. The methodological challenges of such projections are numerous: most often static models are used and their uncertainty is not assessed; valid and comprehensive data to feed into the models are often lacking; and a rapidly evolving environment affects the likelihood of projection scenarios. As a result, the internal and external validity of the projections included in our review appeared limited.

**Conclusion:**

There is no single accepted approach to forecasting physician requirements. The value of projections lies in their utility in identifying the current and emerging trends to which policy-makers need to respond. A genuine gap analysis, an effective monitoring of key parameters and comprehensive workforce planning are key elements to improving the usefulness of physician supply projections.

## Background

**Figure 1:**
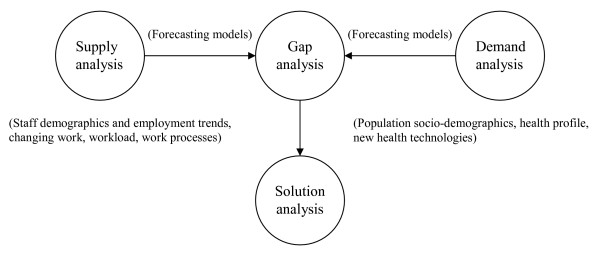
**Main steps of health workforce planning**.

The health care sector is labour-intensive and human resources are the most important input into the provision of health care, as well as accounting for the largest proportion of health care expenditure [1]. Planning human resources for health is the process of estimating the required health workforce to meet future health service requirements and the development of strategies to meet those requirements. Theoretically, it is essentially a two-stage process (Fig. 1), although intermediary steps can be individualized [2].

First, current workforce supply is estimated, and the adequacy of current supply (compared to current requirements) should be assessed. This gap analysis permits identification of current imbalances, provided that the population segment under scrutiny (according to population characteristics, specialty, institution type and location) is precisely defined [3]. Second, a forecast of requirements for professionals is made (usually based on a trend analysis of professional demography and demand for health care), and the optimal workforce size to match those requirements is estimated. Basically, it may be defined as ensuring that the right practitioners are in the right place at the right time with the right skills [4,5].

An oversupply may inflate healthcare costs through a possible supplier-induced demand [6] and may lower quality of health services provided by underemployed physicians, while an undersupply may result in unmet health needs and possible health inequities [7]. Thus, a complex question recurrently lies on the agenda of policy planners: What would be the appropriate number of health professionals needed, given the current national configuration and trends in health services?

To address the question, policy planners have a number of forecasting tools at hand, but the methods, advantages and shortcomings of such tools are not straightforward and not always well appraised. Therefore, this paper has two objectives: (1) to present a typology of existing forecasting approaches, taking the physician workforce planning as an illustrative case; and (2) to analyse methodological challenges of such models and discuss potential paths for improvement.

## Methods

A literature review was carried out in electronic databases Medline-Ovid, Embase and ERIC with the following search terms: health AND (workforce OR manpower OR physicians OR human resources) AND (forecast OR planning OR models). The search was restricted to documents published in Dutch, English, French or Spanish, during the years 1997 to 2007. Documents reporting on physician supply planning in developing countries were excluded. Concrete examples of planning experiences in various countries were analysed.

## Results

### Typology of forecasting models

Four main approaches for physician supply forecast were identified [8].

#### The supply projection approach

Also called the trend model, this relies on physician-per-population ratios and takes into account health care services currently delivered by the total pool of practising physicians. This approach assumes that future requirements for physicians will need to match the volume of services currently provided on a per capita basis. This approach is based on three assumptions: the current level, mix, and distribution of providers in the population are adequate; the age and sex-specific productivity of providers remain constant in the future; the size and demographic profile of the providers change over time in ways projected by currently observed trends [9]. In such models, needs are defined as the necessary inflow of human resources to maintain or to reach at some identified future time, an arbitrary predefined level of service. Thus, the computation of requirements is not based on population health needs.

Although conceptually straightforward, such a model can gain complexity. First, the supply-based model often integrates parameters of demand. Possible changes in demographic features and the delivery system are sometimes factored into the projections. Second, the model is not necessarily based on a simple headcount of providers, but can integrate parameters linked to professional productivity. The model can also serve to create scenarios, such as changes in the skill mix. In such instances, the model is called by some authors a substitution model [10,11]. The service targets approach is similar to the physician-to-population ratio. Requirements are defined on the basis of pre-set health service targets, e.g. staffing required for expansion of facilities [3]. The supply-based approach has been used in Belgium [12], the United States of America [13-17], Australia [18-20], Canada [21] and France [22-25].

#### The demand-based approach

Also called the requirement model or the utilization-based approach, this examines the quantity of health care services demanded by the population. Demand refers here to amounts of the various types of health services that the population of a given area will seek and has the means to purchase at the prevailing prices within a given period. Physician requirements are estimated based on the number and type of projected services and on the physician-per-population ratios in the reference population (population at baseline or benchmarking). This information can be derived from analysis of billing data [26] or from other sources. Generally, the population characteristics considered are limited to age and sex, although other characteristics could/should be incorporated, such as existing market conditions, institutional arrangements, access barriers and individual preferences [27]. Most often also, this approach assumes that physicians are required for all health services that are demanded [28], although the approach can be modified to reflect potential changes to the delivery system. The approach is based on three assumptions: the current demand for health care is appropriate and appropriately met by current level, mix, and distribution of providers; the age and sex-specific resource requirements remain constant in the future; and the size and demographic profile of the population changes over time in ways projected by currently observed trends [9].

Demand can be estimated through at least three methods [29]:

1. The service utilization method: Data on current service utilization serve as a proxy of satisfied demand. This approach is the most commonly used.

2. The workforce-to-population ratio method: A ratio is established between the population (segmented into different age categories) and the requirement for health practitioners. Future projections are based on estimated service need per unit of population and forecast population scenarios. For example, Morgan et al. assessed the adequacy of the oncologist workforce in Australia by using the reference ratio of seven oncologists per million inhabitants. This reference ratio was derived from international benchmarking and expert evaluation [30].

3. The economic demand method: An assessment is made of the current and future social, political and economic circumstances, and how consumers, service providers and employers will behave as a result of those circumstances. Cooper suggested that economic projections could serve as a gauge for projecting the future utilization of physician services [31].

The demand-based approach has been used in various countries such as the United States [14,31-33], Canada [10,11,26] and The Netherlands [34]. As for the supply-based model, models can get quite complex, given the level of precision and of projection adaptability required, as illustrated by the Physician Requirements Model of the Health Resources and Services Administration in the United States [32,35].

#### The needs-based approach

Also called the epidemiological approach, this involves defining and projecting health care deficits along with appropriate health care services. Needs refers here to the number of workers or quantity of services necessary to provide an optimum standard of service and to keep the population healthy. This planning method combines information on the health status of the population with disease prevalence, demographics and appropriate standards of care. The information is essentially provided by professionals.

This approach was used in the United States in the early 1980s, by the Graduate Medical Education National Advisory Committee (GMENAC). Its model used epidemiological evidence for each specialty, modified by professional opinion on the need and appropriateness of care for various conditions to estimate physician need [36]. The following points were considered: incidence rates of specific conditions; percentage of the population with that specific condition who should consult a physician; rate of commonly performed procedures; percentage of procedures that should be performed by a specialist; associated inpatient and office visits per procedure; and productivity estimates/profile of weekly workload.

This approach relies on three assumptions: all health care needs can and should be met; cost-effective methods of addressing needs can be identified and implemented; health care resources are used in accordance with relative levels of needs [9].

An important limiting factor of the needs-based approach is the unavailability of extensive epidemiological data, leading some authors to use an alternative approach based on utilization data. A neat example of this was given by Persaud et al. for ophthalmologists in Ontario [10,11]. The authors used the physician billing claims to measure utilization of services, but also to determine unmet needs and excess utilization (data were adjusted at provincial level for income, education level and Standardized Mortality Ratio).

Moreover, the needs-based approach is more useable when projecting numbers in a specific care specialty, because incidence of the diseases managed within that care specialty can be approximated with more accuracy. An example is the radiologists forecast in Australia. One radiation oncologist is expected to treat 250 new patients per year. The number of radiation oncologists required is thus determined by calculating the number of patients with newly diagnosed cancer during that year and dividing the assumed treatment rate by 250 [30].

#### Benchmarking

This is based on identifying regions or countries that are similar in their demographic and health profiles but are markedly different in their costs and deployment of health care resources. Municipalities and health plans that achieve low levels of deployment of clinically active physicians without a measured loss of patient welfare are considered benchmarks. Those benchmarks are then used as a current best estimate of a reasonable physician workforce active in patient care for planning [37]. Benchmarks can be neighbouring countries or regions within a country, or point estimates from a needs-based approach. Most of the forecasting in the United States during the 1980s and the 1990s, whatever the planning model (supply-, demand- or mixed model), was based on benchmarking. The comparison reference was the staffing pattern in HMOs with adjustments to extrapolate to the general population [33,38].

In benchmarking, the extrapolation methodology is crucial. To draw relevant lessons from a reference model to a specific situation, adjustments are necessary for population demography, population health, patients' insurance, physicians' productivity and health system organization [39]. Obviously, those adjustments are only possible if appropriate information is available.

Our model's typology has been set up to ease understanding (Table 1). In reality, however, projections often combine various models. For instance, in the Netherlands, epidemiological projections were considered along with demographic projections to estimate the evolution of health service demand [34].

**Table 1:** Overview of forecasting approaches

**Forecast strategy**	**Concepts**	**Strengths**	**Limitations**	**Countries**
Supply model	To project the number of physicians required to match the current services given the likely changes in the profession (age, feminization, etc...)	• Can project physician numbers at 10–15 years with accuracy (?)	• Perpetuates current physician-to-population ratio assumed to be adequate • Does not consider the evolution of the care demand	USA [13-17] Australia [18]* Nova Scotia, Canada [21]

Demand model	To project the number of physicians required to match the current services given the likely changes in the demand (mainly population ageing and GDP growth)	• Can anticipate changes in health practices (e.g. new surgical techniques or drugs) and in the health system	• Perpetuates current utilization of services (SID, inappropriate services not addressed) • Assumes that MDs are the main actors and that any care is useful • Does not consider the demand for non curative services (prevention, research) and future trends • Requires huge amounts of data	USA [14,31-33] Canada [10,11,26]

Needs-based model	To project the number of physicians required to provide appropriate health care to the future population	• Rely on a normative approach, i.e. can avoid the perpetuation of existing inequities and inefficiencies • Can include unmet needs in the estimation process	• Requires detailed knowledge of the efficacy of individual medical services for specific conditions • Does not account for technological developments and changes in the organization of health services • The assumption that health care resources will be used in accordance with relative levels of need is not necessarily verified • Ignores the question of the efficiency in the allocation of resources between different sectors of the society	USA [33,36] Ontario, Canada [10,11,50] Australia [30]

Benchmarking	To refer to a current best estimate of a reasonable physician workforce	• Realistic	• Is valid only if communities and health plans are comparable, i.e. adjusted for key demographic, health and health system parameters • Often does not document the extrapolation methodology sufficiently (e.g. unclear criteria for selecting the reference)	USA [13,33,37,40] Australia [30,39]

*: stochastic simulation

The most common mix encountered in the literature associates supply-based and requirement-based parameters, which permits the performance of gap analysis for future years and taking action to make physician supply match requirements. Again, the supply-to-health care utilization ratio at baseline is assumed to be appropriate and serves as a reference for any gap analysis in the future [14,40].

The Effective Demand-based approach is another example of a mixed model. In this approach, the epidemiological principles of the needs-based approach are complemented by economic considerations, i.e. fiscal constraints are integrated in the model [41]. Under this approach, the starting point is to estimate the future size of the economy for which health providers as well as all other commodities are to be funded. This is then used to estimate the proportion of total resources that might be allocated to health care. This approach can in turn be incorporated into an integrated framework. For instance, O'Brien-Pallas has built a dynamic system-based framework (effective demand-based model) that considers: (1) the population characteristics related to health levels and risks (needs-based factors); (2) the service utilization and provider deployment patterns (utilization-based); and (3) the economic, social, contextual, and political factors that can influence health spending [42].

The Effective Infrastructure approach is also based on needs assessment but is complemented by infrastructure considerations. The reasoning is that there is little point in having a workforce greater than the physical capacity of the health system to gainfully employ or use that workforce [43]. Another mixed approach was used by Rizza et al. for endocrinologists in the United States, in which the endocrinologist-to-population ratio computation is based on a Markov-population model including elasticities derived from benchmarking [39].

### Methodological challenges

#### Modeling strategies

Issues relating to human resources are complex in essence, and this complexity will be only partially captured in static models, based on a deterministic approach, such as the majority of the models reviewed above. Even when physician-to-population ratios, population-based rates and utilization-based rates were used as the basis of computerized simulations, these models lacked the capacity to examine the dynamic relationships between inputs and outcomes. There are alternatives to this bounded approach.

First, regression modeling could be a more appropriate approach. Theoretically, regression models can be fit for health workforce projections. Such models allow to adjust for the effect of various parameters and to estimate the importance of each of those parameters to the supply and requirements for health care professionals. It would also be possible to compute confidence intervals around the required numbers. Such models have been used in the United States by Angus et al. [14] and by Lipscomb et al. [44], in Australia [45], and in Ontario by Persaud et al. [10,11]. The difficulty of obtaining accurate data on determinants of services utilization and provision is obvious.

Regression models can also serve as a basis for indirect standardization, as was the case for general-practice workforce modeling in Australia [45]. In that case, however, the regression models were used to identify workforce imbalances at the national level and were not used for forecasting.

A slightly different methodology was used in the United States by Lipscomb et al., who determined physician requirements through empirically based models. Those models were then used to yield estimates of future staffing requirements conditional on future workload, but also to compare current physician staffing in a given setting with system wide norms, i.e. detect under- and over-supply [44].

Second, uncertainty in health projections must be assessed, so that planners can anticipate possible variations and adapt the planning of human resources in consequence. This was rarely the case in the examples presented in the first part of this paper. The two common approaches that can be used are deterministic sensitivity analysis and stochastic simulation.

In sensitivity analysis, a sensitive variable is detected when changes in its input value result in considerable changes in the outcome [46]. In stochastic simulation, the value of input variables is randomly assigned according to their probability distribution and the outcome of the projection will also be a random variable. This process is repeated until a large number of projections have been made. The mean and the variance of the projection's outputs can then be estimated, and the uncertainty of the projections can be quantified by calculating a confidence interval.

Song and Rathwell, who developed a simulation model to estimate the demand for hospital beds and physicians in China between 1990 and 2010, used the two approaches [46]. Their findings indicated that the stochastic simulation method used information more efficiently and produced more reasonable average estimates and a more meaningful range of projections than deterministic sensitivity analysis. They also mentioned that stochastic projection can be used for factors that cannot be controlled by policy-makers, such as population changes.

More recently, Joyce et al. [18], Anderson et al. [33] and Lipscomb et al. [44] have begun testing models for planning resource requirements in health. Simulations can be used to analyze "what if" scenarios – a capability essential for use in health system planning. However, continuously updating estimates is important and simulations can be costly to implement because of their detailed data requirements.

#### Reliability of models

Reliability is defined in the present framework as the capacity of a model to correctly project the health workforce deemed to be adequate at some identified future time. We used three means for exploring models reliability: (1) to compare how a set of models applied to the same setting and the same period produced matching projections (external validity); (2) to examine how projections are sensitive to parameters inserted into the models (internal validity); (3) to confront projections and actual figures (retrospective analysis).

#### External validity

Different models used for projection of health human resource requirements will produce different estimates. Anderson et al., who forecasted the requirement of otolaryngologists in the United States by means of three methods (benchmarking against managed care, demand-utilization modeling and adjusted-needs-assessment modeling) provided a nice example of such a discrepancy [33]. The best estimates for 1994 went from 6611 otolaryngologists with the adjusted-needs approach to 8860 with the demand-based approach, a difference of more than 25%. In 1994, the actual number of otolaryngologists was 7006. Thus, according to the approach, a diagnosis of over- or under-supply could be drawn.

Anderson et al. considered the managed-care approach the most appealing because it reflected the workforce staffing ratios of managed-care organizations that operate efficiently in the marketplace. However, in each of the models, it was possible to show a shortage or surplus of physicians by altering one or more key assumptions.

Persaud et al. also tested the projections yielded by a range of models [10,11]. Their projection of requested ophthalmologists in Ontario for the year 2005 went from 489 FTE (physician/population ratio based on expert recommendation) to 526 ± 16 FTE (substitution model), 559 ± 17 FTE (utilization-based model) and 585 ± 16 FTE (needs-based model). Discrepancies aside, it is noteworthy that the last three models yielded quite close projections.

Interestingly, Politzer et al. reviewed five projection methods for generalist and specialist care requirements in the United States and reached the same conclusion: that different models yielded different figures. But they took advantage of these differences to conduct a type of meta-analysis and to derive requirement bands, instead of one unique requirement figure [47].

The results of projections differ because the models are based on different assumptions. The supply model assumes that existing trends, policies and training positions will be maintained, thus expecting and accounting for no future changes in market factors. The demand model assumes that physician numbers can increase in response to an expected rate of economic growth. The needs-based model assumes that the number of physicians should match the calculated number required to provide adequate medical services to the future population. The first two types of models are based on extrapolation, while the third is based on expert scenarios. The first two types of models aim at projecting a likely future given the current parameters, although some changes can be factored in the models; the third relies on a normative approach. The models also differ in limitations, implications for population health outcomes and resource costs.

#### Internal validity

Whatever the modeling approach, estimates for requirements will not be exact numbers but instead a range of numbers, as several authors have suggested [9,33,46]. Supply-, demand-, and needs-based models are Markov-population models, also called "stock and flow models". Some countries such as Australia, Canada and the United States have used the three types of models alternatively or concurrently.

A Markov-population model can provide a valid projection of the future workforce, provided that the error present in the projection is small and quantifiable, i.e. the inflow and outflow parameters are known with certainty. However, a number of difficulties are also present: (1) small uncertainties in inflow and outflow parameters might result in great inaccuracy; (2) trends, which are often considered to keep on developing infinitely, present plausible limits that must be accounted for; and (3) calculation of statistical confidence intervals is impossible, although there have been attempts to apply those models in a more probabilistic sense [18,33,44].

Although appealing because of its simplicity, benchmarking also presents a number of drawbacks. A similar physician density can provide very different levels of care according to care accessibility, provider productivity, task sharing or prevailing health care delivery model (e.g. the role of a family practitioner can vary greatly across countries). Finally, determinants of population health itself, such as environmental health hazards or lifestyles, can affect the results. For those reasons, it is recommended to use regional benchmarks that are comparable in demographic characteristics and have a similar health system [37].

Attention should be paid to three sets of factors influencing the model's validity: (1) parameter uncertainty, i.e. the quality of available data; (2) the plausibility of projection scenarios, i.e. the likelihood of the underlying assumptions as regards future requirements; and (3) the goodness of fit of the model, i.e. the comprehensiveness of the model and its adjustments for confounding and/or interacting factors.

Data quality is one of the key challenges. Easily accessible clinical, administrative and provider databases are often lacking to conduct complex modeling activities. Even the number of active physicians can be difficult to assess, with important variations between national databases. Moreover, the forecasts usually focus on headcounts, with loose translation into effective workforce. Another example of a loose evidence base is the gender difference of productivity. It is generally estimated that women produce 20% fewer medical services than their male counterparts, an estimate that feeds many models [48]. However, this estimate is not universally applicable and is rapidly evolving, even within a given country.

The likelihood of the underlying assumptions is also an important consideration. In 1998 an undersupply of physicians in Canada was projected for the next 25 years, based on an estimated 31% reduction in the physician-to-population ratio [49]. However, if age and sex-specific needs were to be reduced by 1% per year and average productivity of physicians increased by 1% per year, the physician-to-population ratio would increase by 27% [50]. Therefore, a sensitivity analysis of the models is paramount, for example through stochastic simulation (e.g. Monte Carlo simulation analyses based on bootstrap sampling) [18,44,46]. Re-estimating the dependent variables with subsequent years of data [18] and discussion of clinical plausibility of health demand by a panel of specialists [44] are also means of keeping in line with an evolving reality.

Lastly, the goodness of fit of the model must be assessed. In the models reviewed earlier, adjustment for confounding and/or interacting factors is generally minimal (i.e. for the supply side: profession ageing and/or feminization; for the demand side: population ageing and/or population growth and/or GDP increase). Macroeconometric and microeconometric models of the health care system can be used to draw a more comprehensive view of health workforce planning. However, such models require considerable amounts of data [51].

#### Retrospective analysis

Ultimately, the reliability of the forecasting models can be addressed by analysing the success of past projections in either projecting or modifying the future, i.e. reaching a balance between supplies and requirements. This evaluation is difficult. On the one hand, there are no direct means to assess whether the target was effectively realized [18]. On the other hand, even when the forecast proves correct, the perception of what is an adequate supply/demand ratio can have evolved in the meantime.

It is nevertheless possible to test the realization of projected supply headcounts. We performed the exercise for various countries (Table 2) for which we obtained the human resources statistics for recent years and compared them with the projections previously made by policy planners (Australia [18]; Canada [10,11]; France [25]).

**Table 2:** Projected and actual physician headcounts in selected countries

**Author**	**Country**	**Workforce**	**Models and analysis**	**Base year**	**Time lag**	**Projected**	**Actual**	**Error margin**	**Source of data**
Persaud et al. [10,11]	Ontario, Canada	Ophthalmologists	Multiple regression	2005	10	418 ± 10	387	-5.4%	Ontario Physician Human Resource Data Centre
									
Joyce [18]	Australia	All MDs	Stochastic modeling	2001	2 3	54 294 55 000	56 207 59 004	3.5% 7.3%	Australian Institute of Health and Welfare
									
Doan [25]	France	All MDs	Deterministic	1982	6	180 691	164 667	9.7%	National Medical Council
				1985	9	193 160	184 156	4.7%	National Medical Council
				1988	9	197 406	189 802	4.0%	National Medical Council
				1992	2	185 260	184 516	0.4%	National Medical Council
					7	192 779	196 968	-2.0%	National Medical Council
					12	195 714	211 425	-7.4%	National Medical Council

There was a margin of error in all the projected physician headcounts, and the error size increased with the time lag between projection and assessment. For instance, in Australia, workforce projections have been computed with baseline year 2001 to 2012, on the basis of a supply-based approach [18]. For the first time, stochastic modeling, which employs random numbers and probability distribution, was used. The validity of the modeling has been investigated by comparing the projections with the actual workforce numbers in the early part of the projection period (2002–2003). For 2002 there was a close similarity between the projections and the actual data, but for 2003 the projections were already 3.5% lower than the actual numbers. The reason for this discrepancy was an overestimation of retirement rates (Joyce, personal communication).

## Discussion

### Importance of gap analysis

Planning the health workforce is aimed at having the right number of people with the right skills in the right place at the right time to provide the right services to the right people. It involves comparing estimates of future requirements for and supplies of human resources. However, a major weakness of the examples retrieved in peer-reviewed journals and included in our review was the lack of gap analysis in the reference year, most of the forecasts implicitly making the assumption of an adequate health workforce at baseline. The objective of the projection exercise was therefore to compute the future workforce required to maintain the current equilibrium by taking into account evolving supply and demand trends. However, assessing the adequacy of the workforce and determining the existence of imbalances at baseline is central to workforce planning.

Rizza et al. attempted to apprehend the level of balance between supply and demand at baseline [39]. The authors estimated "current" demand with three indicators: the increase in office visits to endocrinologists in previous years coinciding with a decrease in overall subspecialization rate; the waiting time for initial visit relatively greater for endocrinologists than for other specialties; and an HMO "benchmark" indicating that 12.2% more endocrinologists would be necessary to provide the United States population with health care services equivalent to those provided in the reference HMO. Also noteworthy is that the authors looked at the effect of varying the estimate of the baseline gap between supply and demand on projections.

Morgan et al. accounted for the deficit in radiation oncologists at baseline to compute projected requirements [30]. The specialist deficit was measured by reference to a needs-based estimate. In Australia in 1997 a deficit of 20% in the number of radiation oncologists was reported [30].

Some indicators can be helpful in performing a gap analysis, such as employment indicators (e.g. vacancies rates, growth of the workforce, occupational unemployment rate and turnover rate), activity indicators (e.g. overtime), monetary indicators (e.g. wages), and normative population-based indicators (e.g. doctors/populations ratios) [3]. The AMWAC proposed somewhat similar indicators of undersupply and oversupply (Table 3, adapted from Gavel [43]).

**Table 3:** Indicators of under- and over-supply

***Undersupply***	***Oversupply***
• Doctor provision well below the national average.	• Growth of the workforce well in excess of population growth.
• Underservicing and unmet needs; unacceptably long waiting times; consumers dissatisfied with access.	• Declining average patient numbers; declining average practitioner incomes; insufficient work/variety of work to maintain skills.
• Overworked practitioners; high levels of dissatisfaction with the stress of overwork and inability to meet population needs.	• Underemployment, wasted resources.
• Vacancies, unfilled public positions; employment of temporarily-resident doctors to fill unmet needs; substitution of services by alternative providers.	

However, none of the proposed indicators are unambiguous. For instance, Zurn et al. [3] emphasized that the main limitations of the monetary indicator was that the existence of an imbalance does not necessarily give rise to a wage change as a result of regulations, budget constraints and monopsony power. On addition, wages could increase in consequence of productivity gain or union bargaining power, and not due to an imbalance. Similarly, activity indicators can deteriorate because of a bad management or an inappropriate skill mix, not because of a human resources imbalance. Zurn et al. [3] concluded that relying on a single indicator is insufficient to capture the complexity of the imbalance issue.

It is suggested that a range of indicators should be considered, to allow for a more accurate measurement of imbalances, and to differentiate between short-term and long-term indicators. In addition, further efforts should be devoted to improving and facilitating the collection of data. Moreover, it remains necessary to determine at what level an indicator suggests workforce surplus or shortage, e.g. when a waiting time becomes unacceptable.

### Importance of an effective monitoring of key parameters

We have shown that in most of the reviewed examples, important determinants of supply and demand were not fed into the planning models, most probably because relevant data were not collected and/or not available. The focus to date has very much been on the impact of demographic change on individual health professions, i.e. mainly the effect of an ageing population on the service requirements, and the effect of an ageing workforce on the capacity to meet requirements [50]. As a result, many countries, such as Australia, Canada, France, the United Kingdom and the United States, are balancing from projections of surplus to warnings of shortage with a perplexing frequency.

There is no single accepted approach to forecasting physician requirements [52]. This is a disappointing statement regarding the current utility of planning models. Australia has for years been at the forefront of developing medical workforce planning approaches. However, it has only recently been acknowledged that the Australian workforce planning has so far not taken into account the full range of dynamic variables that are involved, nor accounted for their inherent uncertainty and complex interactions [53]. Subsequently, Joyce et al. have emphasized the importance of an effective monitoring of all key factors affecting supply and demand, i.e. an effective systematic collection of good-quality data to monitor trends over time, as well as the need for a dynamic approach, i.e. to undertake workforce planning in a planned cyclical fashion, with stochastic models to account for the uncertainty inherent in health systems [53].

Table 4 summarizes the difficulties met in collecting such information. An in-depth evaluation of the current situation in human resources for health (HRH) includes an assessment of the current stock of physicians and other health care workers; its composition, gender and age structure; its geographical distribution and its deployment between curative and preventive sectors but also between health care activities and other professional activities (teaching, research, administration, etc.); its activity profile (productivity levels) and working time; its forecasted evolution according to various scenarios; an analysis of the dynamics of the health labour market in terms of entries (including from national training and migration) and exits (deaths, age-related retirement, early retirement); the internal mobility between the public and the private sector, and between the different health care levels (primary care, general hospitals and highly specialized training hospitals).

**Table 4:** Methodological and conceptual issues in forecasting models

**Items**	**Issues**
Model units	• Headcounts do not reflect variation in effective workforce. • FTE measured in working hours can translate into a variable effective workforce. • FTE defined in reference to the most active physician category makes the assumption that the activity level in that category is relevant.
	
Data quality	• Routine data are useful, but provide generally limited information. • Various data sources coexist, with inconsistencies between them. • Qualitative data for in-depth understanding of trends is often lacking.
	
Categories of resources	• Computation of human resources requirements by specialty obviates professional interactions and skill mix. • Assessing skill-mix requirements is a complex task and documentation is often lacking.
	
Supply parameters	• Information other than age, sex and services volume is often unavailable. • Productivity is sensitive to the working and societal environment and is rapidly evolving.
	
Demand parameters	• Assessing the impact of new technologies, emerging pathologies and demographic changes requires a large quantity of data and expertise that are often unavailable. • Level and mode of health care utilization are sensitive to the environment and are rapidly evolving.
	
Modeling	• Deterministic models are likely to generate inaccuracies without providing a means to evaluate them. • Regression modeling with stochastic simulation can be innovative in the HRH field but background is lacking • Regular updating of data is paramount but resource-consuming.

It is also crucial to anticipate the implications of adopting emerging technologies (e-health and innovative treatments including new medicines or day surgeries) and redefining the roles of all available health professionals (distribution of tasks, substitution and delegation). Decision-makers must also review professionals' working conditions and their remuneration (fee-for-service or not) as well as incentives and regulations adopted to attract and retain health professionals in the health sector. How quality of practice would be monitored and ensured is also an important issue to consider. Those choices would have to be validated by the various stakeholders (at the national and regional levels; at the levels of education and training as well as work regulations for professionals) to ensure a reasonable degree of feasibility in their implementation.

International migrations of health professionals in Belgium are a good example of rapidly evolving and challenging key factors to be closely monitored. Since 1997, 100 new yearly incomers were accounted for in the projections, on the basis of a secular trend. The total number of new physicians licensed to practice per year was 700. However, since 2004 there has been a sharp increase in migration influx, with new visas delivered to foreign physicians rising from 138 in 2005 to 430 in 2007.

Before 2004, the inflow originated largely from the neighbouring countries (France, the Netherlands and Germany) and to a lesser extent from Spain and Italy. Since 2004, the larger group of immigrant doctors has come from the eastern part of European Union (Poland and Romania). The enlargement of the European Union since 2004, as well as the implementation of the internal market for services and the mutual recognition of professional qualifications between Member States, favoured the increase.

Another contributing factor has been the limitation of medical trainees (*numerus clausus*) in Belgium, resulting in a decrease in medical assistants and less staff in hospitals. Whatever the causes, this international inflow makes any forecasting of the supply of national health professionals quite difficult and plausibly irrelevant.

It should also be noted that only crude data are available so far, and important parameters such as the proportion of immigrants obtaining a licence to practise in order to further their training (specialization) who will stay in Belgium, turnover rates or activity profiles, are poorly documented. So far, this recent sharp increase in immigrant physicians has not been taken into account in Belgian projections, although it represents more than a 50% excess over the scheduled national numbers and modifies deeply the parameters of the planning.

### Importance of a comprehensive approach

**Figure 2:**
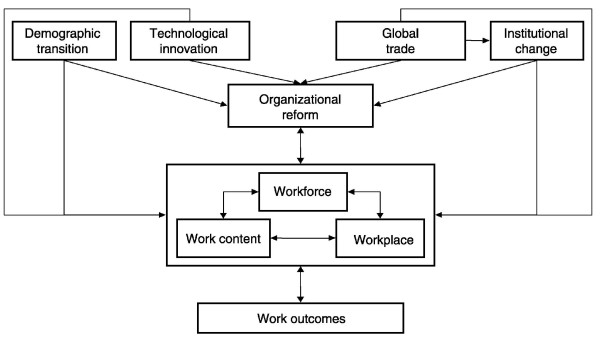
**A framework for analysing future trends in HRH (courtesy of Dubois CA **[55]**)**.

There is no unambiguous "right" number and mix of health professionals, as fundamental societal and institutional dimensions are affecting health workforce production directly and indirectly [52,54]. Dubois et al. recently proposed a neat analysis of factors affecting the health care workforce, as synthesized in Fig. 2[55].

Health provider requirements are determined by broader societal decisions about the level of commitment of resources to health care, organization of the delivery and funding of health care programmes, and level and mix of health care services. We have already underlined the importance of appropriate modeling methods fed with good-quality data. To replace the medical workforce planning in a system-wise approach is also crucial, as other policy initiatives will shape the medical workforce and practice, such as organizational or financial reforms of the health system [55].

However, forecasting the medical workforce is much too often an isolated exercise. Most of the published studies on workforce projections in specific specialties were produced by members of the specialty under consideration. Such a narrow focus may cast some doubt on the validity of the approach and interpretations. Probably the most striking example is given in Shipman et al. [15]. As the authors had observed that the projected expansion was much bigger for the general pediatrician workforce than for the pediatric population, they concluded that "*to maintain practice volumes comparable to today, pediatricians of the future may need to provide expanded services to the children currently under their care, expand their patient population to include young adults, and/or compete for a greater share of children currently cared for by non pediatricians"*.

Such a comprehensive approach is not an easy task for planners. It requires a system-level perspective, integrating medical workforce planning with workforce planning for other health professionals, and with workforce development, service planning and financial planning for the health care system. This broader approach has also been advocated by other authors [41,42,53].

## Conclusion

There is no accepted approach to forecasting physician requirements. Each of the approaches relies on a number of assumptions and limitations that should be acknowledged because of their large influence on the model outputs.

The value of projections lies not in their ability to get the numbers exactly right but in their utility in identifying the current and emerging trends to which policy-makers need to respond. The requirements for health providers are endogenously determined through the political or social choices that underlie the health care system. Only where the social and political choices about the access to and delivery of care are explicit, can scientific methods be used systematically to derive requirements for health care providers in a particular population [50]. However, responsive planning for the future medical workforce remains necessary, as rapid changes are taking place in the supply of medical practitioners and the requirement for their services. Finding this balance requires continuous monitoring, careful choices given the realities of the country, and the use of research evidence to ensure that population health needs are addressed effectively and efficiently [9]. Flexibility, relevance and validity in planning require both ready access to timely information that is accurate and use of appropriate conceptual and analytical techniques.

## Abbreviations

AMWAC: Australian Medical Workforce Advisory Committee; FTE: full-time equivalent; GDP: gross domestic product; GP: general practitioner; HMO: health maintenance organization; HRH: human resources for health; SID: supplier-induced demand

## Competing interests

The authors declare that they have no competing interests.

## Authors' contributions

DR reviewed the literature and drafted the paper. CL and SS critically reviewed the data and contributed substantially to the writing. All authors read and approved the final manuscript.
